# Pre-COPD, PRISm, and COPD in young adults born preterm

**DOI:** 10.1093/annalsats/aaoag182

**Published:** 2026-07-08

**Authors:** Elizabeth F Smith, Callan Lavery, Shannon J Simpson

**Affiliations:** Foundations of Lung Disease, Wal-Yan Respiratory Research Centre, The Kids Research Institute Australia, Perth Children’s Hospital, Nedlands, Australia; Curtin School of Allied Health, Faculty of Health Sciences, Curtin University, Bentley, Australia; Foundations of Lung Disease, Wal-Yan Respiratory Research Centre, The Kids Research Institute Australia, Perth Children’s Hospital, Nedlands, Australia; Foundations of Lung Disease, Wal-Yan Respiratory Research Centre, The Kids Research Institute Australia, Perth Children’s Hospital, Nedlands, Australia; Curtin School of Allied Health, Faculty of Health Sciences, Curtin University, Bentley, Australia

## Introduction

Up to 50% of chronic obstructive pulmonary disease (COPD) cases originate from impaired lung growth and development.^1^ In recognition, the 2023 Global Initiative for Chronic Obstructive Lung Disease (GOLD) update introduced the COPD-Developmental (COPD-D) etiotype, formally recognizing the risk that preterm birth (<37 weeks’ gestation) poses on lifelong lung health.^2^ Preterm-born individuals may have low and/or declining lung function, structural airway abnormalities, and/or respiratory symptoms that persist into adulthood.^3^ Population-based studies report 32-fold higher odds of unspecified COPD diagnosis (*International Classification of Diseases, Tenth Revision, Clinical Modification* code J44.9) in extremely preterm-born (<28 weeks gestation) young adults (18-29 years of age), compared to those born at term.^4^

Like prediabetes or preeclampsia, “pre-COPD” identifies an especially at-risk population of patients who would benefit from closer follow-up and risk management, and provides a critical window for early intervention. “Pre-COPD” describes individuals who do not yet meet spirometric criteria for COPD (typically forced expiratory volume in 1 second [FEV_1_]/forced vital capacity [FVC] ratio <0.70, or below the lower limit of normal), but who exhibit symptoms, radiologic abnormalities, or early functional decline and who are thought to be on a trajectory toward COPD.^5^ Similarly, the preserved ratio impaired spirometry (PRISm) phenotype, characterized by a normal FEV_1_/FVC ratio but reduced FEV_1_, is also frequently associated with transition toward COPD and higher all-cause mortality.^6^ Despite growing recognition of preterm birth as a risk factor for COPD, the incidence of pre-COPD, COPD-D, or PRISm remains unknown in young adulthood survivors of preterm birth.

Here we describe the presence of functional markers of pre-COPD, as well as incidence of PRISM and COPD, in a well-characterized cohort of young adults born very preterm (≤32 weeks’ gestation).

## Methods

Young adults from the longitudinal Western Australian Lung Health in Prematurity (WALHIP) cohort (born 1998-2003, 74% received surfactant, 79% received antenatal steroids^7^) underwent lung function testing (spirometry, plethysmography, and diffusing capacity of the lung for carbon monoxide [Dl_CO_]) according to American Thoracic Society/European Respiratory Society international guidelines, under ethical approval of the Child and Adolescent Health Service Human Research Ethics Committee (RGS815). Z-scores for all tests were calculated using the relevant Global Lung Function Initiative reference equations.^8-10^ Respiratory symptoms were collected via a modified ISAAC questionnaire. Participants were included only if they had valid FEV_1_ and FVC measurements (*N* = 151).

Spirometry outcomes were used to classify participants meeting COPD criteria, defined by an FEV_1_/FVC ratio below the lower limit of normal. COPD severity was categorized as mild (FEV_1_ z-score ≥ −2.51), moderate (−4.1 ≤ FEV_1_ z score < −2.51), or severe (FEV_1_ z score < −4.1). PRISm was defined by a FEV_1_/FVC z-score > −1.64 and FEV_1_ z-score < −1.64. No COPD was defined by both FEV_1_ and FEV_1_/FVC z-scores > −1.64.

We defined pre-COPD by normal spirometry in the presence of (1) hyperinflation, defined by a residual volume/total lung capacity (RV/TLC) z-score >1.64; (2) low Dl_CO_, defined by a Dl_CO_ z-score < −1.64; or (3) FEV_1_ decline, defined by a z-score reduction of at least 0.5 between childhood (4-12 years) and 19-year visits. For participants with multiple childhood visits, the first observation with valid spirometry outcomes was taken.

Clinical factors associated with membership in each group (preterm no COPD, pre-COPD, PRISm, mild COPD, moderate-severe COPD) was assessed via Kruskal–Wallis rank-sum test or Fisher exact test, as appropriate. All analysis was performed using R version 4.5.0.

## Results

Data from 113 very prematurely born participants (mean age, 19.3 ± 1.4 years; gestational age 27.3 ± 1.4 weeks) showed that 42 (37%) met diagnostic criteria for COPD, of whom 35 (83%) were considered to have mild COPD and 7 (17%) moderate COPD (Table 1). A further 7 (6%) participants was classified with PRISm and 14 participants (12%) met criteria for pre-COPD. Among those in the pre-COPD group, 6 (43%) had hyperinflation, 6 had FEV_1_ decline, and 2 (14%) had low Dl_CO_ (Figure 1). There was no overlap between indicators in the pre-COPD group.

**Figure 1:**
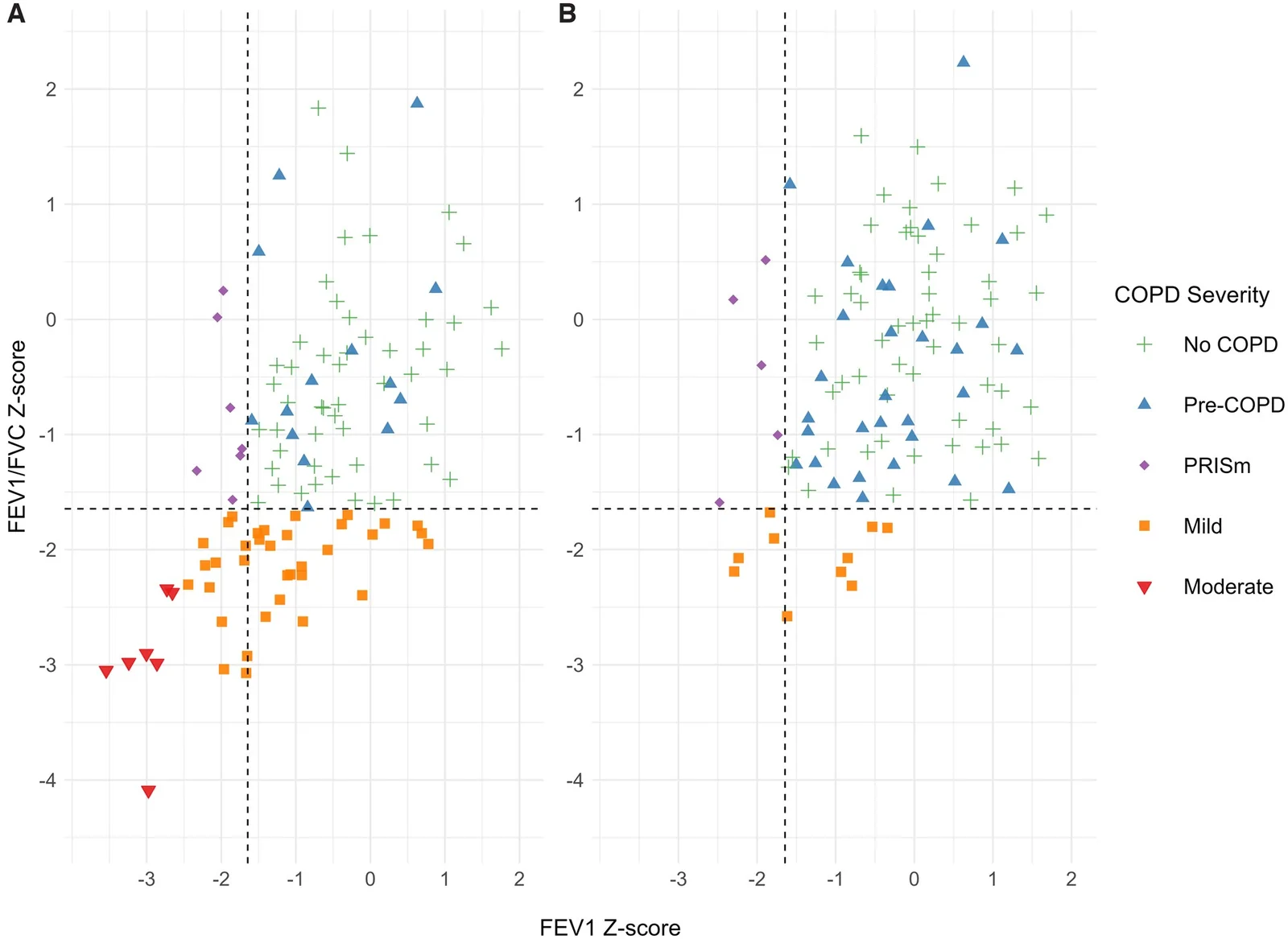
FEV_1_ z-score vs FEV_1_/FVC z-score in young adults born preterm, measured (A) pre-bronchodilator and (B) post-bronchodilator. Dashed lines represent fixed-threshold criteria used to delineate participants with PRISm (upper left quadrant) and COPD (lower left and lower right quadrants). Within the groups without COPD (upper right quadrant), participants with indicators of pre-COPD (hyperinflation, low Dl_CO_, and FEV_1_ decline) are shown. Abbreviations: COPD, chronic obstructive pulmonary disease; Dl_CO_, diffusing capacity of the lung for carbon monoxide; FEV_1_, forced expiratory volume in 1 second; FVC, forced vital capacity; PRISm, preserved ratio impaired spirometry.

**Table 1:** Clinical characteristics and pulmonary function across COPD subgroups in those born very preterm.

Characteristic		Preterm					
	Term	No COPD	Pre-COPD	PRISm	Mild COPD	Moderate COPD	
	(*n* = 38)	(*n* = 50)	(*n* = 14)	(*n* = 7)	(*n* = 35)	(*n* = 7)	
**Demographics**							
** Age, years**	19.44 (1.42)	19.20 (1.32)	19.05 (1.22)	19.50 (1.04)	19.44 (1.55)	19.77 (1.30)	
** Gestational age, weeks**	…	27.57 (25.00, 29.71)	28.21 (25.00, 30.71)	25.71 (24.29, 26.14)	27.00 (25.14, 29.00)	28.00 (24.00, 29.43)	
** Birth weight z-score**	…	−0.05 (0.92)	−0.11 (0.92)	−0.09 (0.71)	−0.08 (0.79)	−0.87 (0.45)	
** Smoking status: current**	2 (5.3%)	2 (4.0%)	1 (7.1%)	0 (0%)	2 (5.7%)	0 (0%)	
** Male sex**	20 (53%)	28 (56%)	10 (71%)	3 (43%)	19 (54%)	4 (57%)	
**Symptoms and diagnoses**							
** BPD diagnosis^a^**	0 (0%)	28 (56%)	8 (57%)	6 (86%)	25 (71%)		5 (71%)
** Physician-diagnosed asthma ever^a^**	4 (11%)	18 (36%)	10 (71%)	2 (29%)	23 (66%)		6 (86%)
** Reported wheeze last 3 months^a^**	0/38 (0%)	1/48 (2.1%)	2/14 (14%)	1/7 (14%)	5/35 (14%)		2/7 (29%)
** Self-reported respiratory hospitalizations^a^**	3/32 (9.4%)	20/50 (40%)	5/14 (36%)	4/7 (57%)	21/35 (60%)		5/7 (71%)
** CT score^a^**	1 (0, 3)	4 (1, 6)	4 (3, 7)	6 (3, 14)	5 (2, 8)		8 (7, 16)
**Pulmonary function**							
** FeNO, ppb**	19 (12, 26)	16 (11, 21)	26 (13, 52)	11 (9, 13)	15 (10, 27)		14 (10, 39)
** FEV_1_ z-score^a^**	−0.01 (1.17)	−0.22 (0.85)	−0.49 (0.83)	−1.94 (0.21)	−1.14 (0.88)		−3.00 (0.31)
** FEV_1_ BDR change^a^**	3 (7.9%)	2 (4.2%)	1 (7.1%)	2 (29%)	15 (43%)		5 (71%)
** FVC z-score^a^**	0.09 (1.07)	0.09 (0.87)	−0.29 (0.89)	−1.51 (0.48)	0.40 (0.97)		−0.85 (1.04)
** FEV_1_/FVC z-score^a^**	−0.19 (1.08)	−0.53 (0.80)	−0.33 (0.99)	−0.81 (0.69)	−2.13 (0.38)		−2.96 (0.58)
**dl_CO_ z-score^a^**	1.43 (0.96)	1.15 (0.86)	0.50 (1.41)	0.22 (1.07)	0.83 (1.09)		0.46 (0.83)
** KCO z-score**	0.88 (0.86)	0.56 (0.89)	0.31 (1.47)	0.75 (1.29)	0.02 (0.86)		0.28 (1.29)
** TLC z-score**	0.21 (0.65)	0.21 (0.77)	0.15 (0.72)	−0.41 (0.78)	0.57 (0.81)		0.16 (1.33)
** RV/TLC z-score^a^**	0.53 (0.76)	0.34 (0.69)	0.97 (1.00)	1.43 (0.88)	0.55 (0.89)		1.47 (0.69)
** FEV_1_ z-score decline^a^**	−0.43 (0.77)	0.25 (0.65)	−0.67 (0.59)	−0.40 (0.72)	−0.46 (0.91)		−1.63 (1.00)

Data are expressed as mean (standard deviation), No. (%), or median (Q1, Q3).

FEV_1_ BDR change is defined as an FEV_1_ increase of >10% relative to the predicted value, following administration of a bronchodilator. FEV_1_ z-score decline is the difference in FEV_1_ from childhood to adulthood. Chest CT scores were derived according to a modification of the scoring matrix by Aukland et al.^14^ where scores are directly related to the number of affected lobes (maximum = 6) except for collapse/consolidation (maximum = 2) with a possible total chest CT score of 50.

Abbreviations: BDR, bronchodilator response; BPD, bronchopulmonary dysplasia; COPD, chronic obstructive pulmonary disease; CT, computed tomography; Dl_CO_, diffusing capacity of the lung for carbon monoxide; FeNO, fractional exhaled nitric oxide; FEV_1_, forced expiratory volume in 1 second; FVC, forced vital capacity; KCO, carbon monoxide transfer coefficient; ppb, parts per billion; PRISm, preserved ratio impaired spirometry; RV, residual volume; TLC, total lung capacity.

a *P* <.05 calculated using Kruskal–Wallis rank-sum test or Fisher exact test as appropriate.

Table 1 shows that the COPD and pre-COPD groups had more respiratory symptoms, with higher rates of physician-diagnosed asthma and wheeze in the 3 months preceding the lung function visit compared with the no-COPD preterm group. RV/TLC z-score, Dl_CO_ z-score, FEV_1_ decline, and chest computed tomography score were also different between the defined groups.

## Discussion

More than 55% of survivors of very preterm birth within this cohort were classified as having COPD-D or COPD precursors (pre-COPD or PRISm) by early adulthood. Despite this high burden of lung disease, coordinated respiratory follow-up programs over the lifespan and published models of care are absent, and clinical management guidelines for prematurity-associated lung disease are based on low or very low levels of evidence.^11^ Furthermore, 85% of adult pulmonologists perceive only moderate to very low awareness of preterm lung disease among their colleagues^12^—highlighting a population that continues to fly under the radar.

The GOLD executive summary advocates for screening spirometry for COPD case finding in those with symptoms and/or risk factors for COPD (eg, >20 pack-years of smoking, recurrent chest infections, or prematurity), where the diagnostic yield for COPD is relatively high.^13^ However, with >2 million babies surviving very preterm birth annually, we urgently need to understand which survivors are at risk of COPD in young adulthood to prioritize those participating in case-finding screening programs. Similarly, once identified, there is no evidence to support appropriate treatment of this population, and randomized controlled trials in patients with “pre-COPD” and in young people with COPD are also urgently required.^13^

The data presented herein should be considered in the context that nearly two-thirds of the WALHIP preterm cohort had a neonatal classification of bronchopulmonary dysplasia (BPD; defined by at least 28 days of supplemental oxygen as assessed at 36 weeks postmenstrual age) due to a deliberate BPD enrichment during recruitment. BPD is a risk factor for lower lung function. Further, although some individuals with mild spirometry impairments move within normal limits following the bronchodilator administration, those with post-bronchodilator spirometry within the normal range still demonstrated a high burden of pre-COPD indicators, and their values remained lower than those of healthy term-born individuals.

There is a high prevalence of asthma diagnoses across both the pre-COPD and COPD groups reflecting the well-recognized overlap between asthma-like symptoms and prematurity-associated obstructive lung disease, although the mechanisms of prematurity-associated lung disease remain poorly understood. Because these classifications are based on physiological markers linked to future fixed airflow limitation, identifying these patterns remains clinically relevant and highlights a spectrum of early airway abnormalities that may progress to chronic obstructive physiology in adulthood. Given the limited mechanistic understanding of preterm-related lung disease and its overlap with both asthma- and COPD-like features, these findings should be interpreted as reflecting a COPD-like obstructive pattern rather than a definitive diagnostic label. Regardless, these data highlight the substantial and still underrecognized impending burden of COPD-like lung disease in those born very prematurely in the postsurfactant era.
